# COVID-19 and distribution centres operations: The impacts and countermeasures

**DOI:** 10.1016/j.heliyon.2023.e18000

**Published:** 2023-07-05

**Authors:** Atif Saleem Butt, Mohammad Alghababsheh

**Affiliations:** a Department of Management, School of Business, American University of Ras Al Khaimah, United Arab Emirates; b School of Business, Department of Business Management, Mutah University, Jordan

**Keywords:** Automation, Case study, Distribution centres, COVID-19, Logistics, Supply chain, Transportation

## Abstract

COVID-19 has wreaked havoc on supply chains. This is particularly true for distribution centres as they struggle to bounce back amid the COVID-19 outbreak. While much literature has recently emerged on supply chain disruption, studies pertaining to the impacts of COVID-19 on distribution centres and the countermeasures taken to mitigate such impacts are elusive and mute. Our study fills this important gap in the supply chain literature. This study employs a multiple-case methodology and conducts 40 semi-structured interviews with senior managers/executives from eight distribution centres in the United Arab Emirates. Our results exhibit that COVID-19 is adversely affecting the distribution centres in at least six distinct ways. For instance, distribution centres are encountering limited staff availability, inventory shortage, destabilized supply chains, excessive inventory, limited capacity and surge in demand. Results also demonstrate six corresponding strategies employed by distribution centres to mitigate the impact. For example, distribution centres enhance warehouse automation, increase hands-on inventory, reshoring manufacturing, use scalable processes and an automation retrieval system, and finally employ a picking strategy. Distribution centres can use the findings provided in this study. Particularly, they can learn how COVID-19 affects them and what corresponding strategies they should adopt to stay strong during this pandemic. This study demystifies its contribution to theory and practice alongside limitations and future research directions.

## 1 Introduction

The impact of the COVID-19 pandemic was first witnessed in the People's Republic of China due to its crucial role in global manufacturing [1]. This is particularly true for the region, Wuhan, the main epicentre of this lethal pandemic outbreak [2]. A recent study by Dun and Bradstreet [2] found that almost 200 Fortune Global 500 firms have a presence in the Wuhan region. It is evident that the impact of COVID-19 is far more sweeping than other types of pandemics in the past. First, this pandemic has affected the entire world, leading to tremendous lockdowns and border closures [3]. Second, additional protocols such as social distancing were introduced to ensure workers' safety, contributing to bottlenecks for distribution centres (DCs) and affecting their operating capacity. Third, the spread of COVID-19 is creating a tremendous amount of ripple effect. For instance, a recent study argued that delays in transportation in one part of the world will have a solid devastating impact globally [4], resulting in massive shutdowns due to the closure of many DCs. Such shutdowns will cause missing, or delay, in supplies of items [5,6].

Notably, DCs are experiencing the worst impacts amid this lethal pandemic compared to other entities in the supply chain (suppliers, warehouses etc.) [3]. Such impacts have affected DCs ability to operate at their fullest potential. For example, a report found that just in the European Union, around 60% of the DCs are operating at less than 30% capacity [7]. Another study concluded that almost 70% of the DCs face some sort of demand disruption due to COVID-19 [8]. Additionally, almost 30% of the DCs have closed for an unanticipated period in China in the first half of 2020 [9]. Moreover, many DCs face unpredictable consumer behaviour (e.g. panic buying), resulting in unprecedented demand, while other industries face demand plummets, leaving DCs in a predicament [3,10]. DCs are also facing a massive backlog of inventory that is just not selling [7]. Furthermore, this paper argues that there will be no short-term fix to meet the growing customer demand. Therefore, DCs would have to think about coping with this unusual situation [11].

The majority of these studies focused on strategies/measures that buyers and suppliers can employ to address the raw material supply process disruptions caused to supply chains amid the COVID-19 pandemic [12,13], while the impacts of the pandemic on DCs’ operations and the measures to address such impacts have received scant attention. The impacts of the pandemic on DCs may vary from one sector to another, making it more cascading and different compared to other entities in the supply chain. For instance, a recent report argues that some DCs are left with excessive inventory amid the pandemic [5]. At the same time, other DCs are at a standstill point while waiting to receive more inventory [1]. In other instances, some DCs are witnessing unprecedented demand in the wake of COVID-19, while other DCs may face demand fall, which negatively impacts the DCs market [7]. A recent study also claims that more research is needed to better understand the impacts of COVID-19 on the management of DCs [1]. Yet, limited evidence exists regarding the most innovative ways DCs can employ to address the impacts caused by COVID-19. This study fills this gap. The following research question is developed to guide this study: H*ow is the COVID-19 pandemic changing DCs operations?*

Precisely, the research objective of this paper is to:

a. To explore the impacts of COVID-19 pandemic on the DCs operations and corresponding countermeasures to address these impacts.
b. To unveil the countermeasures to address/mitigate the impacts.
c. To develop an empirical model from the comprehensive data analysis depicting the different effects of COVID-19 on DCs and linking them further to the most effective countermeasures that they have put in place to streamline their operations.

By answering this question, this study contributes to the literature in three different ways. First, it delves deeply into the impacts of the COVID-19 pandemic on DCs operations. Second, it presents the countermeasures that DCs can employ to meet the challenges amid the pandemic. As such, our study emphases the impact-measure fit concept to handle supply chain disruptions caused by the pandemic. Third, this paper provides strong recommendations to the DCs to turn a problem into a real opportunity and become even more innovative to streamline their business operations during and beyond COVID-19.

This paper is structured as follows: First, this paper unveils the impact of COVID-19 on the DCs. Second, the study demystifies the sample, sampling and data collection process. It also elaborates on the data coding and analysis process and steps to maintain rigour in this qualitative study. Next, the results are presented thematically. Moving on, the theoretical and practical implications of this study are elaborated on. Finally, the paper concludes by discussing the limitations and future research directions.

## 2 Literature review

### 2.1 Supply chain risk management

Despite several attempts, there is no universally agreed-upon definition of supply chain risk. Early supply chain risk management literature attempts to define risk focused on notions from corporate risk management and finance [11]. According to Jüttner [14], risk is the process of identifying, assessing, and mitigating the risks of an organization's supply chain.

Supply chain disruption risk can be caused by both natural and man-made events. Majumdar et al. [15] also noted that environmental risks brought on by natural disasters like floods, earthquakes, and tsunamis can have an impact on supply chain activities by causing disruptions. According to McMaster et al. (2020), transportation is crucial to supply networks. Additionally, a transportation failure affects the lead time, customer expectations, and product life cycle, which may unbalance the supply chain [1]. Furthermore, COVID-19's arrival severely disrupted transportation infrastructure and caused delays in time-definite deliveries [16]. In addition, Majumdar et al. [15] noted that supply chain performance and quality are negatively impacted by machine failure or facility failure in the manufacturing line, which lengthens lead times.

Since COVID-19 originally emerged in 2019, supply chain risk has been the focus of much study. Hosseini and Ivanov (2020) and Sharma et al. (2020) further asserted that pandemic risk is a macro-level risk brought on by the spread of diseases. The operationalization of the supply chain was profoundly impacted by COVID-19 and the Spanish flu in 1918 [17]. According to Chowdhury et al. [16], operational mistakes can result in serious health problems and industrial accidents, whereas [7] discovered that planning risks are brought on by poor production planning, erroneous forecasts, and evaluations of the goods [15] also state that there is a danger of uncertainty when there is a lack of information accessible during a change in the supply or demand side [16] further argued that failure of a supplier to provide the required quality and volume of demand in the desired time results in risks associated with supplier's performance uncertainty.

### 2.2 Role of distribution centres during pandemic outbreaks

Due to the flexible method used at various points in time, the idea of supply chain operations has been continually changing. DCs have a history of participating actively in the supply chain network, even though conventional supply chains have typically focused on attaining cost effectiveness and supply chain excess [18]. There has been a considerable increase in demand for warehouses and DCs after the COVID-19 outbreak as a result of the abrupt switch from large client orders to small customer orders and other alluring services provided by contemporary DCs [11]. As a result, DCs are now crucial to maximizing supply chain operations and meeting the urgent demands of end users. In the end, DCs open the door for product localization and the localization of supplier bases to reflect the requirements and peculiarities of the market and customers [19]. Additionally, DCs are made to fulfill the dynamic requirements of supply chain operations and have shown to be long-term assets [16]. Therefore, the main purpose of DCs is to gather and combine goods from various manufacturers inside one or more enterprises in preparation for product shipping or cross-docking [1].

According to Ref. [3], DCs are storage facilities where a variety of goods in both big and small quantities are gathered from diverse producers. From the same terminal, these cargo are consolidated, sent, and transported to various consumers. Similar to this, the smaller lots that they receive at the DCs are put together, configured in accordance with customers, and then forwarded [20], enabling them to serve the majority of customers with a variety of demands while also saving money. Nevertheless, all DCs now serve as docking hubs where the movement of commodities rather than their storage is the main focus. Govindan et al.'s (2020) discussion of the role of DCs included the following points: (i) fewer storage points; (ii) a greater emphasis on expedient movement of goods rather than their storage; and (iii) an increase in the outsourcing of distribution centres and warehouses.

As a result, DCs engage in highly repetitious, transparent actions that provide a barrier to quantitative measurements [11]. Additionally, the supply chain's whole upstream and downstream activities become more adaptable, which increases the distribution system's resilience to environmental changes. Retailers are made simple by the supply chain's downstream shipment of goods. To enable frequent replenishment at its retail stores in an efficient manner, the supply chain is designed so that stores are built in clusters closer to DCs [21]. However, another challenge that DCs may face after setting and strengthening distribution channel is to move the inventory of finished goods further up the chain either to warehouses or to their company-operated centres instead of stocking with distributors [21]. The relevance of distribution centres in supply chains is well-documented [1,3]), but research on the effects of COVID-19 on distribution centres and the accompanying remedies to meet such issues is lacking. Given that distribution centres support both upstream and downstream supply chain participants, it is clear that their position in a supply chain is crucial. Therefore, any interference with the DC's activities has the potential to affect the whole supply chain.

## 3 Methodology

This study adopts a multiple case study methodology to explore the impacts of the COVID-19 pandemic on DCs operations and their mitigation countermeasures for several reasons. First, case study methodology allows us to explore underdeveloped research topics with limited accumulated knowledge [22]. Second, it enables us to continue an empirical investigation into a limited current occurrence in a setting that is relevant to the actual world [23]. Third, the design of several case studies enables researchers to draw out broad trends from each example [24]. Fourth, using many case studies allows for comparisons, which researchers may use to see whether the results can be confirmed [25,26], boosting the generalizability of the results. Fifth, a case study enables a more thorough and improved understanding of complex phenomena like supply chains, which involve various external actors and functions within the firm [27]. Finally, the case study method aids researchers in gaining a thorough comprehension of a challenging supply chain problem that can broaden knowledge or strengthen what is already understood as a result of earlier research [28].

### 3.1 Study sample and sampling

We interviewed 40 senior managers from eight DCs based in the United Arab Emirates (UAE) - there are over 200 registered DCs trading in the United Arab Emirates. The DCs sampled in this study were catering for the upstream (manufacturers, etc) and their downstream retail customers in the supply chain. These DCs distributed plastic toys to furniture, spare parts, and frozen goods (Table 1 provides further information). Although the role played by DCs vary from sector and region by region within the supply chain, this paper chooses DCs from heterogeneous industries – the DCs were not operated by third-party logistics – to unveil if there is any divergence or convergence in terms of the impacts of COVID-19 as well as the countermeasures employed by the DCs to respond to impacts. It is also imperative to note that the DCs sampled in this study had the full autonomy to enact the countermeasures against the impacts of the COVID-19 pandemic on their accords.

**Table 1:** Profile of interviewees and firms.

No. of firms	Sectors	No. of Employees	Size of the firm	Position of respondents and their codes
Firm I	Spare parts	15000–20000	Large	Customer Service Representative (P1) Vince President (P2) Chief Executive Officer (P3)
Firm II	Spare parts	10000–15000	Medium	Operations Manager (P4) Area Sales Manager (P5) President (P6) Director (P7) Operations Manager (P8) Logistics Manager (P9)
Firm III	Frozen food	5000–8000	Small	Operations Manager (P10) Sales Manager (P11) Logistics Manager (P12) Customer Services Manager (P13) Operations Manager (P14) Transportation Manager (P15) Transportation Manager (P16)
Firm IV	Frozen food	10000–15000	Medium	Accounts Manager (P17 Vice President Operations (18) Chief Executive Officer (P19) Import Manager P20) Sales Manager (P21)
Firm V	Plastic toys	5000–8000	Medium	Senior Vice President (P22) Logistics Manager (P23) Transportation Manager (P24) Accounts Manager (P25) Accounts Manager (P26)
Firm VI	Plastic toys	17000–20000	Large	Area Operations Manager (P27) Sales Manager (P28) Chief Executive Officer (P29) Operations Manager (P30) Operations Manager (P31) Vice President Operations (P32)
Firm VII	Furniture	18000–20000	Large	President (P33) Transportation Manager (34) Customer Services Supervisor (P35) Import Manager (P36)
Firm VIII		5000–8000	Small	Chief Executive (P37) Sales Manager (P38) Operations Manager (P39) Logistics Manager (P40)

To find participants for this study, purposive and snowball selection were used [29]. First, managers were given contact information and the research objectives were explained to the potential participants through a public forum (the name of which is not stated to ensure the anonymity of participants. The interview procedure then started once the knowledgeable respondents (20 participants) were chosen using purposive sampling. As it was exceedingly difficult to find participants on the topic of interest, a snowball sampling methodology was utilized to continue data collection. Twenty individuals were recruited using this method. Table 2 below provides further information below.

**Table 2:** Coding sample of effects of COVID-19 on DCs.

First-order concepts	Second-order themes	Main theme
• Working in shifts. • Extended factory shutdowns. • Limited operating timings of factories.	Limited staff availability	Impacts
• Shortage of raw material on-hand. • DCs are running out of stock slowly. • Limited stock availability.	Inventory shortage	
• Manufacturers located at far-end. • Local manufacturers are operating at a limited capacity. • Delivery of goods taking longer time.	Higher transportation costs	
• Abundance of leftover inventory. • Fluctuation in demand. • Leftover units not selling.	Excessive inventory	
• Limited number of local warehouses. • Limited space to exercise social distancing. • Limited number of decentralized warehouses.	Limited capacity	
• E-commerce orders are all-time high. • Consumers expect fast, accurate and on-time deliveries.	Surge in demand	

### 3.2 Data collection

In total, 40 senior managers from eight DCs were interviewed. Although 15 DCs were sent invitations to participate in the study, only 8 DCs responded back positively due to COVID-19 restrictions in place. Participants provided informed consent to participate. An email containing the project outline was sent to all participants around 6 weeks before interviews. Thirty-two interviews were conducted at the respondents' office, while the remaining 8 interviews were conducted using digital technology due to social distancing policy. Prior to the commencement of an interview, we assured complete anonymity and confidentiality to the interviewees. This helped us elicit a true, accurate and unbiased response on the topic of interest to a great extent.

An interview protocol/guide (see Appendix A) (impacts of COVID-19 on DCs and the countermeasures taken to address these impacts) was emailed along with the research objectives before the scheduled interview dates to begin the data collection process. Interviews were conducted over an extended period of six months – February till July 2021. It is important to note that the pandemic and lockdown situation in UAE during and before the interviews had improved to some extent. Furthermore, although Arabic is an official language spoken in the UAE, all interviews were conducted in English – English is a widely spoken language at the workplace. Interviews with respondents lasted between 60 min and 90 min.

Moreover, all interviews were recorded and transcribed verbatim – saturation was reached after 40 interviews. Also, this study relied on firms' reports, websites, and social media for triangulation purposes [22]. We also conducted a second round of 8 interviews with 8 different participants. The following participants participated in the second phase of interviews: P1, P7, P9, P11, P21, P27, P33 & P38. These were short interviews to check any discrepancies between primary and secondary data. For instance, our interview (primary) data suggested that firms are considering reshoring options within the next 3 to 6 months, while the firm's current policy (through reports) only focused on mitigating transportation costs (not labour costs, production costs etc.). Such cases were brought to the attention of the interviewees to bring more clarity.

### 3.3 Data coding and analysis

Corbin and Strauss [30] propose three different coding types, including open, axial, and selective coding, to code and analyze. All interviews were analyzed on line-by-line basis. Overall, two analysts analyzed the data. A specific code was assigned to each incident or event during initial/open coding. We also used NVIVO to facilitate the coding process and to organize and manage our resources in one place (Welch, 2011). Next, we used axial coding to rearrange data initially divided into categories/codes. Axial coding was also employed to understand how different categories cross-cut and relate to each other. This process resulted in an inter-coder reliability of 92%. Finally, selective coding was used to restrict the coding process to variables of interest and further analyze the relationship between them.

Guba and Lincoln's [31] principles were strictly followed to ensure the trustworthiness of the results: (1) Credibility: participants were asked to review transcripts and provide feedback about any misunderstandings or omissions; (2) Transferability: diverse firms from different industries and participants with differing positions, responsibilities, and from different regions were recruited to ensure transferability. (3) Dependability: data was coded and analyzed by two analysts and interpretation behind assigning each code was discussed between the two analysts; there was 92% inter-rater reliability, which was deemed satisfactory; (4) Confirmability: conclusions backed by quotations, routine data review, and judgmental bias prevention (through two analysts). Table 3 provides a sample of data structure in this study.

**Table 3:** Coding sample of countermeasures taken by DCs.

First-order concepts	Second-order themes	Main theme
• Use 3D printing to bypass the need for carriers. • Using an autonomous version of mobile vehicles. • Using cleaning robots that can pick mobile and collaborative mobile robots.	Increase warehouse automation	Countermeasures
• Develop relationships with extended suppliers. • Open communication lines with second-tier suppliers.	Work with second and third-tier suppliers	
• Manufacture in-house. • Bring the manufacturing process close to the DCs' premises.	Re-shore manufacturing	
• Using software solutions. • Using material handling technology. • Using demand picking system.	Scalable processes	
• Using horizontal carousels directly linked to a floor. • Using vertical carousels to store items swiftly. • Using forklifts in the vertical forms.	Automated retrieval system	
• Picking individual items and adding them to containers. • Using pick and pass picking for individual items. • Using wave picking for individual items.	Picking strategy	

## 4 Findings

Interviews with respondents revealed six impacts of COVID-19 as well as the corresponding countermeasures. They are discussed in Sections 4.1 to 4.6 below. Table 4, Table 5 provides a cross-case comparison of the impact and the corresponding countermeasures.

**Table 4:** Cross case analysis of the impacts of COVID-19 on DCs.

Distributors	Limited staff availability	Inventory shortage	Higher transportation costs	Excessive Inventory	Limited capacity	Surge in Demand
Firm I	Limited working hours of staff	Limited stock availability	Production facilities are closed	Orders becoming obsolete in warehouses	Limited storage capacity	Consumers expect fast, accurate and on-time deliveries
Firm II	Production facilities are operating at limited capacity	DCs running on a marginal basis	Higher labour costs	Abundance of leftover inventory	Limited number of local and decentralized warehouses	E-commerce sales are all-time high
Firm III	Staff working in different shifts	Shortage of essential and non-essential items	Production facilities are closed	Wild fluctuation in orders	Limited number of local warehouses	Consumers expect fast, accurate and on-time deliveries
Firm IV	DCs running with limited capacity	Shortage of raw material	Limited direct approach of DCs to manufacturers	Orders becoming obsolete in warehouses	Limited storage locations	Online orders are reaching an all-time high
Firm V	Staff working in different shifts	Limited stock availability	Slow shipping and logistics process	Abundance of leftover inventory	Limited number of local and decentralized warehouses	E-commerce sales are all-time high
Firm VI	Extended factory shutdowns	Shortage of essential and non-essential items	Higher transportation costs	Orders becoming obsolete in warehouses	Limited space for social distancing	E-commerce sales are all-time high
Firm VII	Limited staff availability to curb COVID-19	DCs running on a marginal basis	Limited logistics services	Wild fluctuation in demand	Limited storage capacity	Online orders are reaching an all-time high
Firm VIII	Limited working hours of staff	DCs running on a marginal basis	Limited direct approach of DCs to manufacturers	Wild fluctuation in orders	Limited space for social distancing	Consumers expect fast, accurate and on-time deliveries

**Table 5:** Cross case analysis of countermeasures to mitigate the impacts of COVID-19 on DCs.

Distributors	Increase automation	Increase hands-on inventory	Reshore manufacturing	Scalable processes	Automated retrieval system	Picking strategies
Firm 1	Use automated guided vehicles	Escalating the space of warehouse and higher capacity to fit in new-inventory	Manufacture as close as possible to the DCs	Flexible order picking strategy	Using horizontal carousels directly linked to a floor	Picking individual items and adding them to containers
Firm II	Use autonomous mobile robots	Work with second-tier and third-tier suppliers	Manufacture in-house and as close as possible to the DCs/warehouses	Use a combination of material handling technology and software solutions	Using vertical carousels to store items swiftly	Using pick and pass picking for individual items
Firm III	Combination of automated guided vehicles and mobile robots	Escalating the space of warehouse and higher capacity to fit in new-inventory	Manufacture in-house	Using software solutions to manage DCs	Adhering to the operator's command	Using parallel picking for individual items
Firm IV	Use autonomous mobile robots	Work with second-tier suppliers	Manufacture closer to the warehouses/DCs	Flexible order picking strategy	Using forklifts in the vertical forms	Using wave picking for individual items
Firm V	Use automated guided vehicles	Work with second-tier and third-tier suppliers	Manufacture in-house	Using software solutions to manage DCs	Using horizontal carousels directly linked to a floor	Picking individual items and add them to containers
Firm V1	Combination of automated guided vehicles and mobile robots	Escalating the space of warehouse and higher capacity to fit in new-inventory	Manufacture closer to the warehouses/DCs	Use material handling technology	Using vertical carousels to store items swiftly	Using pick and pass picking for individual items
Firm VII	Using the autonomous version of mobile vehicles	Work with second-tier suppliers	Manufacture as close as possible to the DCs	Use a combination of material handling technology and software solutions	Adhering to the operator's command	Using parallel picking for individual items
Firm VIII	Using cleaning robots that can pick mobile and collaborative mobile robots	Escalating the space of warehouse and higher capacity to fit in new-inventory	Manufacture in-house and as close as possible to the DCs/warehouses	Flexible order picking strategy	Using forklifts in the vertical forms	Using wave picking for individual items

### 4.1 Impact I: limited staff availability

Respondents argued that COVID-19 infected many workers globally, leading firms, particularly on the front lines, to implement strict new preventive measures. Furthermore, the spread of the virus has been strong enough to warrant the shutdown. However, many businesses are struggling to operate with some additional health measures. Eventually, the best way to keep the employees healthy is to reduce the number of employees in the building at a given time. Unfortunately, such strategy directly thwarts the production process and it becomes even more critical than ever for DCs to operate at full capacity. DCs also face myriads of challenges in adjusting inventory counts and making sufficient space for work in progress. Respondents also highlighted challenges about delivering orders on time and speeding up the entire process during this critical condition.

*Our staff is working in shifts and warehouses, encountering extended shutdown as coronavirus has spiked over the last few months. Our staff is at serious risk of being affected. We have had to reduce our timings. It is quite unfortunate, but I guess we do not have an option at this stage. The health of our staff is our utmost priority (P1)*

#### 4.1.1 Countermeasure I. increased warehouse automation

Respondents argued that they are relying on the automation process in the DCs to address the staff shortage issues. For instance, they are using the main autonomous versions of mobile vehicles such as autonomous mobile robots and automated guided vehicles in DCs. Automated guided vehicles help transport goods throughout a warehouse of manufacturing environment without human intervention. They also assist in rail navigation, magnetic tape navigation, or wire-guided navigation to move around a facility, which means they follow a fixed path. These machines can also perform repetitive jobs that are dangerous for the staff due to COVID-19. It is important to note that Firm I Firm II, Firm VII and Firm VIII have already implemented these two main types of automation. In contrast, the remaining firms have partially automated their operations (they are using either automated guided vehicles or autonomous mobile robots). Furthermore, Firm I, Firm II, Firm VII and Firm VIII noted that it takes a significantly longer time to build a new automated system — up to one year or more

*We have robots picking and packing great success. Using robots has also helped us maintain social distancing between workers. We are also using the autonomous versions of mobile vehicles to reduce the chances of spreading viruses. We also found this technology very easy to set up, implement as compared to other automated systems. We also found the use of cleaning robots that can pick items are very helpful in automation process*(P7)

### 4.2 Impact II: inventory shortage

Narratives revealed that manufacturing firms opted for lean manufacturing as their best practice to meet the inventory shortage before the pandemic arrived. Respondents further prompted that receiving goods in faith and just-in-time for manufacturing has kept their inventory cost down. Just-in-time also facilitated the reduction in costs and they were able to utilize more space efficiently. However, COVID-19 has put them in a very frustrating situation. The lean manufacturing strategy they rely on has completely left their inventory in a massive shortfall. Lean manufacturing has even put the production process to a complete halt. Furthermore, the just-in-time strategy is also not working effectively and resulting in a tremendous inventory shortage. Consider the quote below supporting this notion.

*Well, we are facing a substantial shortage of raw material on hand. Our DCs are running out of stock slowly. We also used to rely on lean manufacturing before the arrival of this pandemic. This practice has been working extremely well and kept the costs of our inventory down, but this lethal pandemic has badly affected our lean practices and we have literally no inventory left (P4)*

#### 4.2.1 Countermeasure II: increase hands-on inventory

While lean manufacturing has remained their best practice, COVID-19 has changed the balance between the just-in-time inventory and the safety stock in hand. Respondents further argued that to reduce inventory shortage in the foreseeable future and ensure that production facilities continue to operate at par, they ensure buffer inventory in hand. Two respondents also emphasized that they are not entirely sure yet how much inventory would be needed as it depends on myriads of factors, but the overall inventory need had increased. Having enough inventory in hand will potentially escalate the space of the warehouse and higher capacity issues. Still, they will need a space to manage the additional inventory eventually to make sure that they do not run of stock of at least essential items. DCs are thoroughly working with their secondary suppliers to ensure that they have enough inventory in place to avoid just-in-time and avoid working with manufacturers using a lean manufacturing strategy.

*We are working with secondary suppliers (and even third-tier suppliers) to keep inventory on hand and prevent further inventory shortages. We have also asked our key manufacturers to avoid practising lean as this jeopardize our stock level. Well, this strategy has its own repercussion but it will bring some sort of reprieve to the DCs in terms of managing inventory. This is a need of the time, I guess (P16)*

### 4.3 Impact III: higher transportation costs

Interviews revealed that COVID-19 has disrupted the global supply chains in myriad of ways, resulting in higher transportation costs for the distributors across the country. Firms are also facing higher freight costs. As a result, many manufacturing plants have come to a complete halt. Also, DCs do not have any direct approach to the manufacturers. There are limited efforts to reduce the risk of any future shortages and potential shutdowns. They have to work with manufacturers located in the high-end region or far located manufacturers within the country.

Furthermore, securing the future of supply chains is becoming somewhat elusive with a global pandemic in place. The nearby manufacturing sites cannot produce more with less, and it is becoming cost-competitive as they have to rely on manufacturers operating in farside areas. Narratives unveiled a dire need to create a useable space for reshoring markets. Consider the quote below.

*We are facing higher freight as our local manufacturers have shut down their operations. We have to work with the manufacturers located at the far end of the country. We never witnessed such massive disruptions to the supply chains. There is a substantial shortage of production, and manufacturing plants are just literally closed. There is rather no approach or strategy in place to reduce the risk of shortages or shutdowns (P21).*

#### 4.3.1 Countermeasure III: reshoring manufacturing

Many respondents employed reshoring strategy to bring down the supply chain costs and bring more stability. Respondents narrated that during this uncertain time, this strategy appears to be very beneficial. At first, reshoring is helping firms to bring the cost down to a great extent. Narratives reveal that due to higher transportation and labour costs, limited access to the required material, and travel restrictions, the idea of reshoring is speeding up. They told that they shifted the manufacturing to different regions and further assessed their process around the major issues, such as how they will manage the flow of material, manage labour efficiencies, and use all space available. Also, shifting manufacturing operations to other regions will increase the control on the supply chains to a great extent and further prevent future crises. It will further save the significant ocean and air freight transportation costs. The following quote testifies to this:

*We are reevaluating our processes and considering nearshoring. For example, we recently decided to bring the manufacturing process all the way to our premises or at least as close to the home. This will help us save supply chain associated costs and bring more stability to supply chains (P33)*

### 4.4 Impact IV: excess inventory

Interestingly, the discussion revealed that DCs have been experiencing wild fluctuation in demand amid COVID-19 with limited resources in place to handle this issue. Respondents contended that the orders have reduced and they are essentially left with an abundance of inventory that is just not selling at any cost. The excess inventory, particularly for non-essential items such as furniture and plastic toys, is taking up valuable space. Moreover, DCs do not have any mechanism or successive planning to predict the fluctuating demand, resulting in a higher leftover inventory. Additionally, they do not have any labour requirements to cope with the sudden shift in demand or any other strategies to fully ascertain how to handle the wild fluctuation in customer demand.

*We have excessive stock in hand because we are so puzzled by this weird consumer behaviour, creating panic regarding what to really order. We just do not know how to handle this change in demand. Many units are just not selling (P38)*

#### 4.4.1 Countermeasure IV: employ scalable processes

Respondents suggested that although they are left with plenty of inventory due to varying patterns in demand, they have implemented some strategies to cope with this issue. For instance, they are employing a scalable process to manage any fluctuations in demand. Two respondents stated that their firms are now using a combination of technologies related to material handling and additional software solutions. This is helping DCs to deploy a very flexible strategy when it comes to picking orders. Furthermore, it is also allowing them to accommodate peaks in the order demand easily. For example, one respondent stated that their warehouse has demanded picking systems and processes, which has increased picking speeds within 24 h. DCs also have to adjust their labour requirements and the automation process to handle the demand fluctuation. Consider the following quote.

*It is surprising that we still got plenty of stock left while other competing firms have apparently no inventory in hand. Nevertheless, we use a demand picking system and different material handling technologies to manage our warehouse operations. It also helps us in picking the right quantity of orders (P15)*

### 4.5 Impact V: limited capacity

Interestingly, COVID-19 has resulted in a limited capacity at DCs and they are now left with increased inventory on hand. They are also facing additional challenges about meeting the increased demand and notably strict precautionary measures to maintain social distancing. Further varying factors have compelled DCs to develop more storage locations and capacity. Respondents also revealed that they are searching for ways to maximize floor utilization and optimize storage capacity. They narrated that both are equally important to manage the DCs' limited capacity. Two respondents also said that COVID-19 has resulted in higher demand for essential and non-essential items. They are experiencing significant difficulties and there is a large growth of certain manufacturing items. DCs are expeditiously running out of space and struggling to keep up with the wild fluctuations in demand. Consider the quote below:

*We are facing a rise in demand, and businesses cannot cope with this sudden change. We are also marginally operating with a limited number of local warehouses. We also have limited space to exercise social distancing. So what can I really say? We are literally left with no options on how to increase the capacity. It is very problematic (P23)*

#### 4.5.1 Countermeasure V: use an automated retrieval system

Results state that DCs are relying on technology to change or expand the capacity of DCs. In particular, respondents pointed to the use of automated storage and other equipment. For instance, two participants pointed out that they use horizontal carousels directly linked to an overhead or floor mounted rack from a complete loop range. This automated storage system has the potential to eliminate any unproductive and unneeded travel and search time and directly deliver the product to the operator. Two additional respondents argued the use of vertical carousels (compared to horizontal ones) are helping them to store items more quickly and safely to the positioned counters of work. They also adhered to the operator's command and further reduced the time needed to walk and search for the items in the DCs. Three additional respondents highlighted the use of an automated retrieval system to manage capacity within their DCs. For instance, they pinpointed to vertical forms that automatically locates stored trays from the columns. It also retrieves such trays from the columns and passes them on to the operators at a pick window, thus fully eliminating the need to travel and Stock Keeping Unit (SKU) each item in the DCs. The following testifies to this:

*We have different systems in place, such as horizontal carousels directly linked to a floor and vertical carousels to store items swiftly. Essentially, they are helping us to reduce the space and therefore eliminating the need to think about expanding our warehouses. Such additional space has helped us even store more products in the same amount of facility and the exponential growth in the number of total Stock Keeping Unit stored in the warehouse (P3)*

Another respondent stated that:

*We have implemented warehouse scalability as an option to keep track of inventory in the warehouse. It also helps us keep note of the work in the warehouse, thus allowing us to accommodate the warehouse's growth and the excess inventory, which is essentially no longer needed (P28).*

### 4.6 Impact VI: surge in demand

Discussion with many respondents revealed that COVID-19 is primarily responsible for a sudden surge in demand and they are facing extreme challenges in responding to a wild fluctuation in demand. They stated that their DCs are not ready to cope with online orders. They further argued that they are hopeful that consumers would be potentially eager to return to the stores for shopping, which might help them anticipate the demand accordingly; however, they also pointed out that the boom in e-commerce will not subside when the impact of COVID-19 is minimal. Moreover, many shoppers are returning the products they bought online initially instead of personally visiting the stores amid the COVID-19 outbreak. At the same time, respondents narrated that over 50% customers are expected to continue to shop online until the situation settles down to some extent. Consider the following quote testifying to this:

*We are going through a very difficult time when it is just literally impossible to keep our hands on the demand for certain essential products. E-commerce is at every time high than ever expected. Online shopping is booming up and almost 65% of our shoppers are now buying pretty much all products online, and there is a minimal visit to the store during COVID-19. This trend is likely to continue for – I’m not sure – some time (P 20).*

#### 4.6.1 Countermeasure VI: use picking strategy

While the DCs are struggling to meet the recent surge in demand, some respondents pointed to using a split-case picking strategy to manage online orders and fluctuations in increasing demand. They further narrated that the perspective of online and in-store shopping has entirely changed in the past year. E-commerce demand is high and shows no signs of slowing down – at least in the short run. Consumers are now expecting much faster and accurate on-time delivery in this era. Therefore, they have to employ the strategy of split-case picking. Respondents highlighted that such split case picking is all about picking individual items and adding them directly to the shipment containers to be sent out to customers in a timely manner. This saves the consumers' time and when they open that case, they find their order adjusted. This process is very different from picking full cases and sending them to the retailers. Many respondents also reported picking and passing and wave picking to manage online orders. However, split-case picking is labour intensive and challenging to perform and must be exercised with caution. Consider the quote below:

*We are adjusting our operations to ensure it is smooth running. For instance, our staff are now picking individual items and adding them to containers and further opting for pick and pass picking for individual items. We also use wave picking for individual items in certain instances. It allows us to better manage the demand at peak and helps us grow our operations and better manage our e-commerce within the second line of our business (P11)*

## 5 Discussion of empirical findings

The following research question was developed at the outset of this study: How is COVID-19 changing DCs operations? Upon pursuing this goal, the result reveals that COVID-19 is changing DCs in six distinct ways. First, distribution centres are encountering limited staff availability and therefore enhancing the automation process. Second, DCs are working with second-tier suppliers (besides first-tier suppliers) to address the inventory shortage. Third, DCs are working with manufacturers to engage in reshoring to minimize the higher transportation costs. Fourth, DCs are left with excess inventory, which is just not selling. Consequently, they are employing scalable processes to sell the unsold inventory. Fifth, DCs are employing an automated retrieval system to address the issues of limited capacity, and, finally, DCs are executing different picking strategies (e.g., pick individual items, using parallel picking) to address the wild surge in demand.

First, results of this study indicate a production shortage due to strict security measures and limited staff availability onboard at the DCs. Yet, this paper found that many DCs have responded well to the declining production by partially or fully automating their warehouses (using robots etc.). Results also state that warehouse automation played an extremely pivotal role in increasing the production capacity amid COVID-19. Our findings provide empirical support to recent literature on the role of automation in DCs. For instance Ref. [10], argued that COVID-19 could encourage production units to rely on automation processes to subsidize the employees' productivity.

Second, DCs can work with manufacturers employing just in time and lean manufacturing but with caution as this can escalate the distribution centre's space and further raise capacity issues. Moreover, results suggest that firms can revert to lean manufacturing and just-in-time inventory once the pandemic is over. These findings concur with existing literature, suggesting that lean and just-in-time manufacturing would be affected amid the COVID-19 outbreak as DCs might be reluctant to work with them [1,21]. Third, this study found that COVID-19 has created an unprecedented ripple effect, spreading upstream to the downstream supply chain. In particular, the manufacturing sector has been disturbed, adversely affecting the logistics operations (trucks shortages, limited routes etc.) and the DCs operations. This paper found that many DCs are constantly negotiating with their manufacturers to either re-shore or nearshore manufacturing. Precisely, many DCs have successfully negotiated with their manufacturing to temporarily shift their operations near the DCs to shorten the delivery times between the two entities. Recent studies have also emphasized that DCs should encourage manufacturers to consider reshoring to speed up the delivery process [3,16,32]. Thus, this paper empirically shows that reshoring could be an excellent option for DCs to streamline their internal business operations.

Fourth, some DCs reported increased leftover inventory, not selling due to a wild fluctuating demand. Recent literature points out this impact as well. For instance Ref. [4], argued that COVID-19 could result in varying demand patterns, affecting suppliers' decision-making when ordering essential items. While this study [4] notices that firms may be left with unsold inventory, resulting in potentially higher costs, it does not unveil what DCs can do to overcome this issue. This paper contributes to this body of literature. This paper found that DCs employ picking strategies and cutting-edge technology to ascertain the demand fully.

Fifth, our results suggest that DCs have limited capacity due to the social distancing rules and strict safety measures, and DCs are struggling to arrange for enough storage capacity. These findings are supported by existing literature. For instance Ref. [5], ran a simulation study and argued that safety protocols to mitigate the effect of COVID-19 would compel DCs to work at a minimal capacity. This notion supports the findings of this study [11] also pointed out that DCs can use forklifts in vertical formats to better manage their capacity. However, this paper digs deeper into this phenomenon and provide additional ways that DCs can increase their capacity. For instance, findings suggest that DCs can use an automated retrieval system besides relying on forklifts. They should use horizontal and vertical ventricular to eliminate unproductive and unneeded travel and search time and deliver the products directly to the operator.

**Fig. 1:**
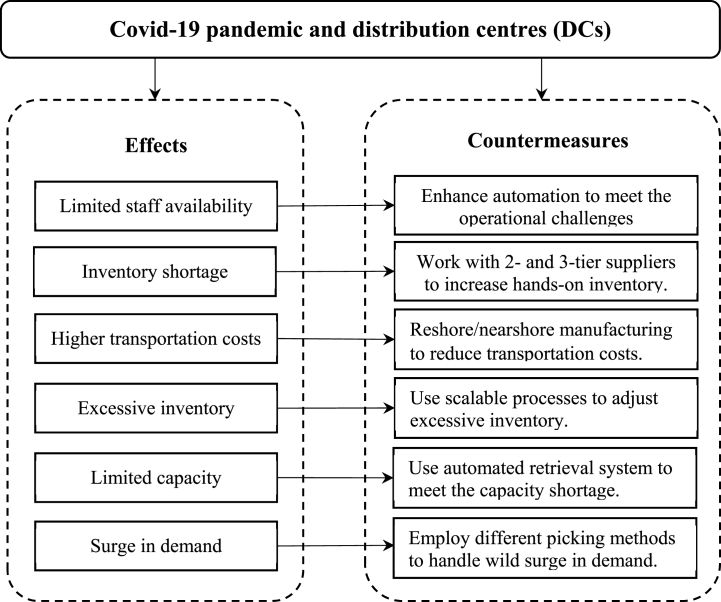
Initial casual mode of effects of COVID-19 on DCs and corresponding countermeasures.

Sixth, results point to a surge in demand amid COVID-19. Many DCs are not prepared to handle online orders. Furthermore, many DCs have no separate departments to handle online orders and e-commerce planning. These findings are new to the supply chain disruption literature from the perspective of pandemic outbreaks. To better manage the surge in online orders, DCs are using a split-picking strategy in the form of pick and pass picking and wave picking - to only pick the orders their direct suppliers need. These findings contradict the existing literature. For instance, Karmaker et al. (2021) argued that split case picking might not effectively handle demand amid COVID-19 outbreak. However, this study presents a different empirical perspective and suggest that a picking strategy is beneficial for DCs to handle the fluctuating demand. Based on the above discussion, we hereby propose an empirical model (Fig. 1) as below:

## 6 Implications for firms

This study has implications for practice. First, it highlights the impacts of the COVID-19 pandemic on DCs operations and the corresponding countermeasures to streamline their business operations amid the pandemic outbreak. While many DCs are struggling to cope with this challenging situation, there lies an opportunity for them to make the best out of this unusual situation and bounce back even stronger. It is also worth mentioning that some of the practices presented as DCs countermeasures are derived from their discourse with their customers upstream and downstream, and these are not stand-alone decisions at the distribution centre level.

Second, while it is evident that DCs are struggling to manage the unprecedented surge in demand, it is recommended that DCs/warehouses use a split-packing strategy. In other words, they can manage the demand well if they pick individual items and add them directly to the shipment containers to be sent out to customers in a timely manner. Indeed, this will help suppliers save plenty of time as well. However, this study proposes that this process is very labour-intensive and requires plenty of effort on the worker's part. Moving on, the impact of COVID-19 is creating an enormous ripple effect and disrupting the entire supply chain from manufacturing to the end consumer. However, DCs can still operate effectively if the manufacturing firms open up temporary production facilities nearby, reducing travel time between two connected parties. This could also bring more stability to the entire supply chain and mitigate the higher transportation and labour costs. Such reshoring will also help improve material, labour, and efficiencies between different entities in the supply chain.

Third, results suggest that DCs are operating at a reduced capacity. Respondents' stories thoroughly pointed out the issues of managing inventory due to limited space available. Yet, employing an automated retrieval system to manage the space within the warehouse effectively. Therefore, DCs should seriously consider horizontal carousels to decrease unnecessary travel and search time. It will also help workers deliver the product to the operator directly and within the minimum time possible. A vertical carousel can also prove effective as it can help deliver items expeditiously and safely to work counters.

Finally, as COVID-19 has reduced staff availability at the work floor, resulting in production shutdowns, firms should automate their warehouses from scratch. Furthermore, DCs can employ different automation methods such as autonomous guided vehicles and autonomous robotic vehicles. They can be a suitable replacement for workers in the DCs in various forms such as product picking.

## 7 Limitations and future research directions

This study comes with certain limitations. First, it relies on a relatively small sample size of 40 respondents. Hence, caution should be taken while generalizing its results to a broader population. Second, the interviews were conducted in one interval focusing on the cause and effect relationship. Finally, although we had a second meeting with some respondents to discuss the discrepancies between primary and secondary data, future research should collect data longitudinally. Having data on a longitudinal basis can help firms capture an even more accurate picture of the impacts and countermeasures. Furthermore, the context should be considered as the results of this study are obtained from a Middle Eastern country (UAE), which may not be transferable to other countries.

This study also opens additional avenues for future research. First, the results of this study should be tested through surveys for authentication. Second, some respondents argued that a picking strategy effectively manages the demand and comes with demerits. Therefore, future research could delve into how picking strategy affects warehouse operations and labour productivity adversely. This will help us understand how effective the use of the picking strategy would be amid COVID-19. Third, while lean manufacturing is discouraged during this uncertain situation, some respondents argued that ignoring lean manufacturing can uniquely impact the supply chain. Future research should address this question. How can avoiding lean manufacturing affect the supply chains in the COVID-19 era? Are there any unique negative impacts?

Fourth, respondents pointed out that, though reshoring can serve as an essential means of streamlining the flow of resources between the DCs and manufacturers, it can bring additional issues. Hence, the research explores the opposing sides of reshoring the production vicinities to give a more balanced perspective on the importance of reshoring in the wake of pandemic outbreaks. Finally, data was collected from participants based in one country – UAE. This country is enriched with financial resources and relies on the cutting-edge and latest technology to meet the challenges of COVID-19. It would be interesting to ask a similar question in developing countries to ascertain the countermeasures' impact and efficacy as developing nations usually have limited resources.

While COVID-19 has disrupted global supply chains, the impact of COVID-19 on the DCs is going to have long and lasting effects compared to other actors in the supply chain. Furthermore, the impact of COVID-19 on DCs varies from industry to industry. For instance, some DCs are left with excessive inventory (particularly of non-essential items such as plastic toys and furniture) during the pandemic. At the same time, other DCs are at a standstill point waiting to receive more inventory (food industry etc.). In addition, some DCs are witnessing unprecedented demand in the wake of COVID-19, while other DCs are facing demand fall, leading to a negative impact on the DCs market.

Finally, some impacts and countermeasures (limited staff availability, increased use of automation) apply to other entities in the supply chain and production facility). Yet, the majority of the impacts and countermeasures unveiled in this study are unique to DCs only (scalable processes, automated retrieval system, picking strategy etc). Therefore, future research should focus on the application of the strategies developed by DCs to other supply chain member firms.

## Production notes

### Author contribution statement

ATIF SALEEM BUTT: Conceived and designed the experiments; Performed the experiments; Analyzed and interpreted the data; Contributed reagents, materials, analysis tools or data; Wrote the paper.

Mohammad Alghababsheh: Analyzed and interpreted the data; Contributed reagents, materials, analysis tools or data; Wrote the paper.

### Data availability statement

The authors do not have permission to share data.

## Declaration of competing interest

The authors declare that they have no known competing financial interests or personal relationships that could have appeared to influence the work reported in this paper.

## APPENDIX A

Interview protocol

a. How long have you been working for this firm?
b. Could you please tell me about your key stakeholders?
c. Could you please discuss how COVID-19 is changing the landscape of distribution centres?
d. Could you please elaborate on the current plans in place to manage disruptions to your operations?
e. May we ask if the impact of COVID-19 on suppliers or manufacturers you work with is having any direct/indirect impact on your internal/external operations?
f. How do you view the long-term future of distribution centres once the pandemic is over?
